# The effect of local perturbation fields on human DTI: Characterisation, measurement and correction

**DOI:** 10.1016/j.neuroimage.2011.12.009

**Published:** 2012-03

**Authors:** Siawoosh Mohammadi, Zoltan Nagy, Harald E. Möller, Mark R. Symms, David W. Carmichael, Oliver Josephs, Nikolaus Weiskopf

**Affiliations:** a Wellcome Trust Centre for Neuroimaging, UCL Institute of Neurology, University College London, London, UK; b Magnetic Resonance Unit, Max Planck Institute for Human Cognitive and Brain Sciences, Leipzig, Germany; c Department of Clinical and Experimental Epilepsy, UCL Institute of Neurology, University College London, London, UK; d MRI Unit, National Society for Epilepsy, Chalfont St Peter, UK; e AMRIG, UCL Institute for Neurology, Queen Square, London, UK; f Imaging and Biophysics, UCL Institute for Child Health, Guilford Street, London, UK

**Keywords:** Diffusion tensor imaging, Eddy current correction, Mean diffusivity, Diffusion-weighted images, Local perturbation fields

## Abstract

Indices derived from diffusion tensor imaging (DTI) data, including the mean diffusivity (MD) and fractional anisotropy (FA), are often used to better understand the microstructure of the brain. DTI, however, is susceptible to imaging artefacts, which can bias these indices. The most important sources of artefacts in DTI include eddy currents, nonuniformity and mis-calibration of gradients. We modelled these and other artefacts using a local perturbation field (LPF) approach. LPFs during the diffusion-weighting period describe the local mismatches between the effective and the expected diffusion gradients resulting in a spatially varying error in the diffusion weighting **B** matrix and diffusion tensor estimation. We introduced a model that makes use of phantom measurements to provide a robust estimation of the LPF in DTI without requiring any scanner-hardware-specific information or special MRI sequences. We derived an approximation of the perturbed diffusion tensor in the isotropic-diffusion limit that can be used to identify regions in any DTI index map that are affected by LPFs. Using these models, we simulated and measured LPFs and characterised their effect on human DTI for three different clinical scanners. The small FA values found in grey matter were biased towards greater anisotropy leading to lower grey-to-white matter contrast (up to 10%). Differences in head position due to e.g. repositioning produced errors of up to 10% in the MD, reducing comparability in multi-centre or longitudinal studies. We demonstrate the importance of the proposed correction by showing improved consistency across scanners, different head positions and an increased FA contrast between grey and white matter.

## Highlights

► Measurement of local perturbation fields (LPFs) on three different scanners. ► LPFs are, e.g., diffusion-gradient non-linearities/mis-calibration. ► LPFs bias fractional anisotropy (FA) and mean diffusivity (MD) maps. ► Correction improves accuracy, FA contrast and comparability between scanners. ► Relevant for group, longitudinal or multi-centre studies.

## Introduction

Diffusion tensor imaging (DTI) offers the possibility of estimating quantities related to the brain's microstructure (Basser et al., 1994; Le Bihan et al., 1986; Pierpaoli and Basser, 1996; Turner et al., 1990). DTI indices, such as the mean diffusivity (MD) or the fractional anisotropy (FA), are often used in research studies to correlate function with structure (Powell et al., 2006; Vernooij et al., 2007) as well as in clinical DTI studies to identify diseased areas in white matter (WM) microstructure (Deppe et al., 2008; Gupta et al., 2005; Horsfield and Jones, 2002; Kleffner et al., 2008; Meinzer et al., 2010) or even in grey matter (Keller et al., in press). DTI is susceptible to imaging artefacts, which can vary significantly between magnetic resonance imaging (MRI) scanners. Artefacts will perturb the diffusion-weighted (DW) images, bias the estimation of the diffusion tensor and reduce the comparability of DTI indices. Artefact characterisation, measurement, and correction are crucial steps for the wider application of DTI particularly to enable multi-centre and longitudinal studies as well as clinical application.

DTI relies heavily on the performance of the scanner's gradient system. The generated gradient for diffusion weighting may differ from the requested one in magnitude and direction due to eddy current (EC) fields (e.g. Haselgrove and Moore, 1996; Jezzard et al., 1998), gradient nonuniformities (e.g. Bammer et al., 2003), mis-calibration of the gradient amplitude (e.g. Nagy et al., 2007), and concomitant field terms (e.g. Meier et al., 2008). These deviations from the expected (linear) gradient performance and also other effects biasing the diffusion weighting, such as cross terms between diffusion-weighting gradients and imaging gradients (e.g. Neeman et al., 1995), can all be modelled by a general local perturbation field (LPF). In a first order approximation, the LPF can be described by a matrix (LPF matrix), which accounts for the deviation between the theoretically assumed and effectively applied diffusion gradients. The LPF matrix is spatially dependent. An LPF affects the DW images differently during the course of the DTI pulse sequence. Perturbations during the echo planar imaging (EPI) readout period cause image distortions, which can be retrospectively corrected if the LPF matrix is known. LPFs during the diffusion-weighting period lead to a spatially varying error in the **B** matrix (i.e. the experimental design matrix relating DW images to the diffusion tensor (Mattiello et al., 1997)) and bias the diffusion-tensor estimation. Bammer et al. (2003) addressed this bias using simulated and phantom data. They proposed a retrospective correction that uses a matrix model to account for the effect of the LPFs due to gradient nonuniformities, provided that the gradient nonuniformities are known (e.g. provided by the vendors). Another correction method, which can be applied to any sequence or scanner, even if the LPFs are unknown, was suggested by Nagy et al. (2007). They measured deviation from the known true diffusion coefficient of a water phantom, which should ideally be isotropic and spatially invariant. The deviations of the measured water diffusion coefficient from the known one can be modelled by LPFs which include, but are not limited to, the gradient nonuniformities. Nagy et al. (2007) used this information to adjust the amplitudes of the diffusion gradients in read, phase and slice direction to yield accurate diffusion measurements in the isocenter. This approach corrects for global effects only, i.e. each gradient direction is corrected by one empirical scaling factor. In a follow-up study, Nagy et al. (2009) extended their approach to correct the measured apparent diffusion coefficient (ADC) on a voxel-by-voxel basis in post processing. However, unlike Bammer's approach, their method was not constrained to a specific model and thus less robust to slight variations in experimental conditions. Up to now, the effects of LPFs on the diffusion tensor have only been studied on phantom data (Bammer et al., 2003) or in tractography studies Nagy et al. (2007). Thus, it has not been investigated how diffusion tensor index maps in different brain tissue types (e.g. FA values in grey and white matter) are affected by LPFs during diffusion weighting.

Here, we estimate LPFs and correct human DTI data by combining both previous approaches. Following Nagy et al. (2007) we measure the deviation from isotropic diffusion in a water phantom. Our approach then is to use a physically constrained model to robustly estimate the LPF and, following the matrix model of Bammer et al. (2003), we correct for the effect of the LPFs. We first describe the theoretical framework of our model. We then test the robustness of our model, with respect to variation in LPFs and measurement noise, using Monte Carlo simulations. Finally, we illustrate the importance of the proposed method by estimating the LPFs, correcting the DTI measurements, showing their effects on human DTI index maps, and comparing the results on three different scanners.

## Theory

### The tensor model for diffusion

The diffusion tensor **D**, as introduced by Basser et al. (1994), is linearly related to the apparent diffusion coefficient, ADC_*i*_:

(1) $${\text{ADC}}_{i}(r)=B_{i}:D(r)=\sum_{k,l=1}^{3}\;{\left(B_{i}\right)}_{\mathrm{kl}}{\left(D(r)\right)}_{\mathrm{kl}}\text{,}$$

where *r* is the position vector and the elements of the B matrix are given by:

(2) Bikl=bgk,igl,ifork,l=1,…,3

where *b* summarises the extent of diffusion sensitization as described by Mattiello et al. (1997), $g_{i}$ is a unit vector $g_{i}=G_{i}/|G_{i}|$ pointing along the direction of the *i*th diffusion-weighting gradient, $G_{i}$ (*i* = 1,..., *N*_DG_, *N*_DG_: number of diffusion gradient directions). In this model, the ADC is calculated from the logarithm of the ratio of the measured non-DW and DW signal divided by the *b* value (Basser et al., 1994).

### The perturbed diffusion tensor

According to Eq. (1), the measured ADC not only depends on the diffusion tensor, D, but also on the B matrix, i.e. on the magnitude and direction of the applied diffusion gradients. However, the effectively applied diffusion gradient, $G_{i}^{*}(r)$, usually deviates from the theoretically assumed spatially linear diffusion gradient, $G_{i}$, due to local perturbation fields (LPFs) such as ECs, gradient nonuniformities, concomitant fields, gradient mis-calibrations, and cross terms between imaging and diffusion gradients. As a result, the estimated diffusion tensor might be perturbed, $D^{*}(r)$, if in Eq. (2) the theoretically assumed diffusion gradient vectors, $g_{i}$, are used to calculate the B matrix (see also (Bammer et al., 2003)):

(3) $${\text{ADC}}_{i}^{*}(r)=B_{i}^{*}(r):D(r)=B_{i}:D^{*}(r)\text{,}$$

where ${\text{ADC}}_{i}^{*}(r)$ is the measured apparent diffusion coefficient.

### The linear perturbation model

For small and linear perturbations, i.e. in first-order perturbation theory (Arfken and Weber, 2001), the deviations between theoretically assumed, $G_{i}$, and effectively applied, $G_{i}^{*}(r)$, diffusion gradients can be described by an LPF matrix, *Σ*(*r*), correcting the magnitude and direction of the diffusion-weighting gradients:

(4) Gi∗(r)=(I3+Σ(r))Giand

(5) gi∗(r)=(I3+Σ(r))gi

where $I_{3}$ is the identity matrix and *Σ*(*r*) is a smooth function of space and comprises time-averaged LPFs in first order. Note that $G^{*}$ and $g^{*}$ do not account for higher-order perturbations.

Given the approximation in Eq. (5) and Eq. (2), the perturbed B matrix reduces to:

(6) $$B_{i}^{*}(r)\approx B_{i}+2\Sigma^{+}(r)B_{i}=L^{\Sigma }(r)B_{i}\text{,}$$

with

(7) $$L^{\Sigma }(r)\equiv I_{3}+2\Sigma^{+}(r)$$

and *Σ*^+^ being the symmetrical part of the LPF matrix *Σ*^+^ = (*Σ* + *Σ*^T^)/2 (see Eq. (A.3) in Appendix A). It follows from Eq. (7) that $L^{\Sigma }(r)$ is positive definite for small perturbations. Therefore, $L^{\Sigma }$ will be subsequently denoted as the *LPF ellipsoid*. Moreover, it follows from Eqs. (3) and (6) that the symmetric part of the LPF matrix, *Σ*^+^, is sufficient to correct the LPF related perturbations of the diffusion tensor to first order:

(8) $$D^{*}(r)=L^{\Sigma }(r)D(r)\text{,}$$

### LPF ellipsoid ($L^{\Sigma }$) and the isotropic*-*diffusion limit

In the isotropic-diffusion limit, i.e. for voxels with small FA values in GM or cerebrospinal fluid (CSF), the perturbed diffusion tensor is proportional to the LPF ellipsoid:

(9) $$D^{*}(r)\approx DL^{\Sigma }(r)\text{,}$$

It is known that small FA values can be strongly biased by perturbations (Pierpaoli and Basser, 1996; Skare et al., 2000). Eq. (9) shows that within the isotropic-diffusion limit the maps of indices of the LPF ellipsoid, such as the principal axis (*e*_∥_^*Σ*^), the fractional anisotropy (${\text{FA}}^{\Sigma }$), and the trace of $L^{\Sigma }$, directly relate to the perturbations within the respective index maps of the human–brain diffusion tensor. In other words, e.g., the trace map of $L^{\Sigma }(r)$ identifies biased regions in a standard diffusion MD map that is estimated from human DTI data and affected by LPFs. For brevity, this paper presents results for the trace and FA maps of $L^{\Sigma }$ only.

### Estimating the LPF ellipsoid ($L^{\Sigma }$) and matrix (*Σ*)

We estimated the LPF matrix by acquiring DTI data of a water phantom, for which the diffusion coefficient, *D*_*w*_, is known to be isotropic. If follows from Eq. (3) and Eq. (9) that the measured ADCs of the water phantom are related to the LPF ellipsoid $L^{\Sigma }$ as:

(10) ADCi∗(r)≈DwLΣBi.

The procedure to estimate the LPF ellipsoid $L^{\Sigma }$ and matrix *Σ* is described in Appendix B. Briefly, it can be divided into two steps: in the first step, we estimated the $L^{\Sigma }$ ellipsoid (Eq. (10)) on a voxel-by-voxel basis using the water phantom DTI data (see Eq. (B.1)). From the LPF ellipsoid $L^{\Sigma }$ one can directly determine the LPF matrix *Σ* using Eq. (7). In the second step, we used the $L^{\Sigma }$ ellipsoid matrix elements as data points for estimating the spatial dependency of the LPF matrix elements under the constraint that the LPF can be described by 3rd-order spherical harmonics (see Eq. (B.6)).

## Methods

### Subject, data acquisition and processing

We acquired DTI data sets from a healthy subject (male, age 34) with written informed consent according to the guidelines of the local ethics committee at three different scanners (DTI1, DTI2 and DTI3). DTI1 was acquired using a 3T TIM Trio scanner (Siemens Healthcare, Erlangen, Germany), operated with an RF body transmit coil and a 32-channel receive-only head coil. DTI2 was acquired using a 3T Achieva TX scanner (Philips Healthcare, Best, The Netherlands), operated with an RF body transmit coil and a 32-channel receive-only head coil. DTI3 was acquired using a 3T Signa HDx scanner (General Electric, Milwaukee, WI, USA), operated with an RF body transmit coil and an 8-channel receive-only head coil. The Stejskal–Tanner diffusion pulse sequence scheme was used on all three scanners (Stejskal and Tanner, 1965). The MRI protocol details are summarised in Table 1.

On any given scanner a different protocol was used, which was identical for both phantom and human measurements (see Table 1). All data were collected at the isocentre. To test the LPF effects when scanning at different positions within the gradient coil, the DTI3 data were acquired a second time, shifting the subject by 3 cm. The DTI data sets were preprocessed by correcting for motion and affine whole-brain EC image distortions during EPI readout (Mohammadi et al., 2010). To increase the signal-to-noise ratio (SNR) for the estimation of the LPFs, the phantom DTI images were smoothed using an isotropic Gaussian 5-mm full-width-at-half-maximum (FWHM) kernel. All analysis steps were performed using SPM8 (http://www.fil.ion.ucl.ac.uk/spm) and in-house software written in MATLAB (version 7.11.0; Mathworks, Natick, MA, USA).

### Analysis I: Precision and robustness to measurement error

We assessed how well the proposed linear model estimated LPFs in a simulation of a water phantom DTI data set. The procedure for testing the precision of the linear model was divided in four steps.

*Step 1*: 100 LPFs were simulated as 3rd-order spatially-dependent spherical harmonics (see Eq. (B.3) and (Arfken and Weber, 2001)). The coefficients of the spherical harmonics were randomly chosen with constant probability from the interval [− 1, 1] and the simulated LPF was normalised to a peak-to-peak variation of 0.1, i.e. 10% of the theoretically assumed diffusion gradient.
*Step 2*: The perturbed DW images were calculated using the simulated LPFs and Eq. (3). The simulated diffusion coefficient *D*^sim^ is chosen in such a way that the ratio between the non-DW and the DW images is 1/5, if the perturbation is set to zero and the *b*-value is equal to *b* = 1000 *s*/mm^2^.
*Step 3*: Gaussian-distributed noise was added to the perturbed DW and non-DW images, resulting in a realistic SNR of 50 for the non-DW image and 10 for the DW images. Next, the non-DW and DW images were smoothed with a Gaussian smoothing kernel (5-mm FWHM), and the resulting ADCs were recalculated.
*Step 4*: The LPFs were estimated from the smoothed ADCs using the proposed model (see section Estimating the LPF ellipsoid LΣ and matrix Σ).

To quantify the precision of the estimation, we calculated for each trial the normalised mean difference between simulated and estimated LPF matrix elements *δε*_*ij*_ (i.e. the modulus of the difference between simulated and estimated LPF matrix elements divided by the average of the modulus).

### Analysis II: Estimating and correcting LPFs

The LPF was estimated based on the proposed model from water phantom DTI data resulting in three LPFs (one for each data set): *Σ*_DTI1_, *Σ*_DTI2_, *Σ*_DTI3_. The estimated LPF matrix was then used to correct the measured diffusion tensor for the effects of LPFs on phantom and human data. To assess the importance of the off-diagonal elements of the LPF matrix, we performed two different corrections:

• *Correction 1*: Only the diagonal elements of the estimated LPF matrix were used, which account only for the LPF gradients that were parallel to the applied diffusion gradient.
• *Correction 2*: The entire estimated LPF matrix was used including off-diagonal elements.

#### Correcting phantom DTI data

The performance of the proposed correction method on the DTI data of the phantom was checked by calculating the FA of the measured data (${\text{FA}}^{\text{meas}}$) as well as that of the corrected data (correction 1: ${\text{FA}}^{\text{cor}1}$ and correction 2: ${\text{FA}}^{\text{cor}2}$). In particular, it was investigated whether the perturbed FA images matched better and were closer to the expected FA = 0 when the DTI data were corrected. For this purpose, we calculated the histogram of the FA maps of the three DTI data sets.

#### Correcting human DTI data

The performance of the proposed correction method on human DTI data was investigated by comparing the FA images for each DTI data set, calculated before and after correction. To identify regions in diffusion FA and MD maps that were expected to be most strongly affected by the LPF, we also calculated the FA and trace of $L^{\Sigma }$ for each DTI data set. We hypothesised that the contrast between low FA and high FA regions (i.e. typically GM and WM) would be increased by the correction. To estimate the improvement in grey-to-white matter contrast of FA maps, we segmented the non-DW images (Ashburner and Friston, 2011) and used the thresholded GM and WM probability maps (*p* > 0.85) to calculate the contrast before and after correction in a region-of-interest (ROI). As a measure of contrast we defined: ${\bar{\Delta \text{FA}}}_{\mathrm{cont}}\equiv FA_{\mathrm{ROI}}^{\mathrm{WM}}-FA_{\mathrm{ROI}}^{\mathrm{GM}}$ and calculated the FA histogram in the GM and WM masks. We also investigated how different head positions would interact with the LPF induced errors. To this end, we compared the measured and corrected MD maps for different head positions shifted in the z direction (*Δz* ≈ 3 cm).

## Results

### Analysis I: Precision and robustness to measurement error

**Fig. 1:**
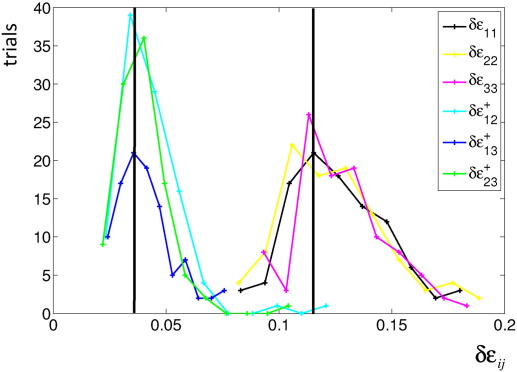
Histogram of the normalised mean-difference between simulated and estimated LPF matrix elements *δε*_*ij*_ (*i*, *j* = 1,..., 3), a measure for the precision of the estimation. The error is higher for the diagonal elements (≈ 12*%*) than for the off-diagonal elements (≈ 4*%*).

We used a histogram of the normalised mean difference between simulated and estimated LPF matrix elements to determine the precision of the linear model (*δε*_*ij*_, with *i*, *j* = 1, …, 3, see Fig. 1). The matrix elements of the LPF matrix were estimated with approximately 4% (off-diagonal elements) and 12% (diagonal elements) error.

### Analysis II: Estimating and correcting LPFs

**Fig. 2:**
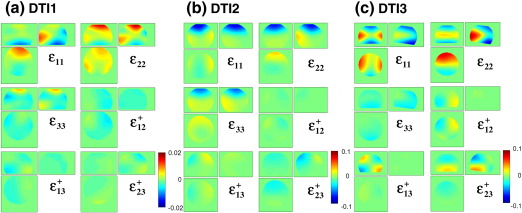
The six LPF matrix elements estimated from phantom data: (a) DTI1, (b) DTI2, and (c) DTI3. Note that the limits of the color map differ by a factor of 5 between DTI1 and DTI2/DTI3.

Fig. 2 shows the estimated LPF matrix elements for: DTI1 (a), DTI2 (b), and DTI3 (c). The amplitude of the estimated LPF matrix elements of the data set DTI1 was significantly smaller (maximum ≈ 2%) than the amplitudes of the LPF matrix elements of the data sets DTI2 and DTI3 (maximum ≈ 10%). The amplitude of the perturbation field was minimal in the centre of the phantom for all matrix elements.

**Fig. 3:**
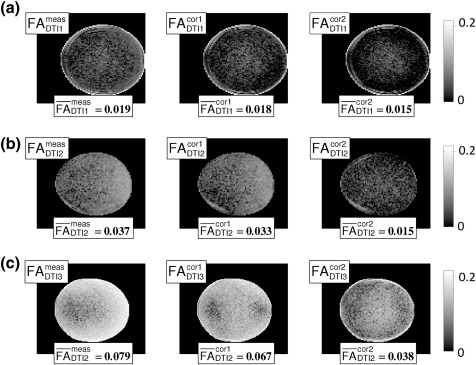
FA images of an isotropic water phantom before and after correction for different acquisitions as well as the averaged FA value ($\bar{\text{FA}}$) over the whole phantom: (a) DTI1, (b) DTI2, and (c) DTI3. From left to right: FA of measured DTI data, FA of corrected DTI data using solely the diagonal elements of the LPF matrix, and the whole estimated LPF matrix. The FA images are depicted in log-scale to emphasise the improvement after correction. The FA values within the phantom decrease after correction as expected in the case of isotropic diffusion.

After correction, the FA of the water phantom was effectively reduced everywhere in the phantom (Fig. 3), in accordance with isotropic diffusion in water. This effect was most pronounced if the whole LPF matrix (right column) was used for correction as compared to using only the diagonal elements of the LPF matrix (middle column).

**Fig. 4:**
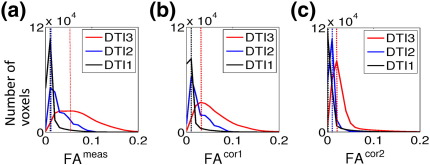
FA histogram plots comparing the FA in the three water phantom data sets: (a) using the measured FA images (FA_DTI1, DTI2, DTI3_^meas^), (b) and (c) the corrected FA images (FA_DTI1, DTI2, DTI3_^cor1^ and FA_DTI1, DTI2, DTI3_^cor2^). After correction the position of the maximum of the FA histograms is closer to zero and the shape of the distribution is narrower, indicating an increase in consistency and accuracy. The improvement was bigger when off-diagonal elements of the LPF matrix were included (FA_DTI1, DTI2, DTI3_^cor2^).

The FA histograms in Fig. 4 show that for the corrected FA maps more FA values were closer to zero and their distribution was more narrow than for the measured FA maps, i.e. the bias towards higher FA values was always reduced. Furthermore, the maxima of the distribution of the FA histograms of the two data sets were most similar if the whole LPF matrix was used for the correction (Fig. 4c), suggesting a better comparability between corrected DTI data from different MRI scanner.

**Fig. 5:**
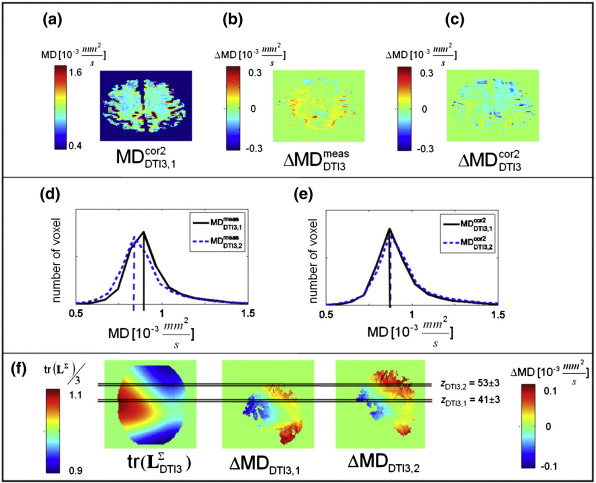
Effect of repositioning on MD images of human DTI using the DTI3 scanning protocol (DTI3,1 and DTI3,2) acquired at different positions along the z direction (*Δz* ≈ 3 cm). From top left to bottom right: (a) MD of corrected DTI data (MD_DTI3, 1_^cor2^) at position *z*_DTI3, 1_ (see (f)), (b,c) MD difference image of repositioned data sets before (*Δ*MD_DTI3_^meas^ = MD_DTI3, 1_^meas^ − MD_DTI3, 2_^meas^) and after (*Δ*MD_DTI3_^cor2^ = MD_DTI3, 1_^cor2^ − MD_DTI3, 2_^cor2^) correction, (d,e) histogram for measured and corrected MD of data set DTI3,1 (black) and DTI3,2 (blue) in slab of slices highlighted in (f) (slab size ≈ 1.7 cm, i.e. 7 slices, position of central slice *z*_DTI3, 1_ = 41 and *z*_DTI3, 2_ = 53), (f) sagittal view of (from left to right): trace of the LPF ellipsoid tr(**L**_DTI3_^*Σ*^) estimated from water phantom DTI data set, MD difference maps of corrected and uncorrected DTI data with different position in z direction (*Δ*MD_DTI3, 1_ = MD_DTI3, 1_^cor2^ − MD_DTI3, 1_^meas^ and *Δ*MD_DTI3, 2_ = MD_DTI3, 2_^cor2^ − MD_DTI3, 2_^meas^). A repositioning error of 3 cm leads to an error of more than 0.1 × 10^− 3^ mm^2^/*s* in MD (d) that can be corrected using the proposed LPF correction method (e). Note that for better visualization the MD images in Fig. 5b and 5c were realigned and smoothed.

Fig. 5 shows that the correction increased the comparability of MD maps. Variation of the positioning of the subject by 3 cm (Fig. 5f) led to a change in MD of about 10% (Figs. 5b,d). Using the proposed correction method, the error was significantly reduced and the similarity of the distribution of MD values in brain tissue increased (Fig. 5c,e).

**Fig. 6:**
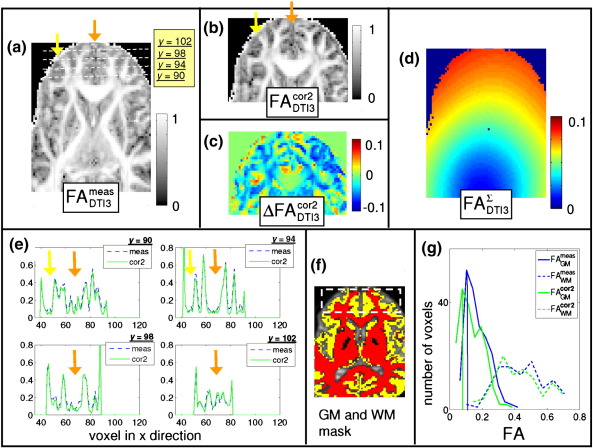
FA images of human DTI before and after correcting data set DTI3 for LPFs. From top left to bottom right: (a) FA of measured DTI data FA_DTI3_^meas^; (b) FA of corrected DTI data (FA_DTI3_^cor2^); (c) difference image between corrected and measured FA image ΔFA_DTI3_^cor2^= FA_DTI3_^cor2^-FA_DTI3_^meas^; (d) FA of the LPF ellipsoid FA_DTI3_^*Σ*^ estimated from the water phantom DTI data set; (e) profiles of FA values along the dotted line in (a); (f) non-DW image overlaid by segmented grey matter (yellow) and white matter (red) masks. The grey and white matter masks are used to calculate the averaged FA contrast within the highlighted region in (f); (g) histogram of white matter and grey matter FA within the highlighted region of the masks in (f). The FA images (a,b) are depicted in log-scale to emphasise the improvement after correction. The small FA values in grey matter decreased after correction (blue regions at anterior part of the brain in (c)). Regions within the diffusion FA maps that were most affected by the LPFs (see (c)), were identified by high-FA-value regions of the LPF ellipsoid (red regions in (d)). Note the inverse color scale for (c) and (d): negative *Δ*FA is blue whereas high FA^*Σ*^ (red) leads to negative *Δ*FA (blue).

Fig. 6 shows that the GM/WM contrast ${\bar{\Delta \text{FA}}}_{\mathrm{cont}}$ was greater for the corrected (Fig. 6b) than for the measured (Fig. 6a) FA images in areas strongly affected by LPFs. Within the dashed region in Fig. 6f the GM/WM contrast improved on average by more than 10%, i.e. from ${\bar{\Delta \text{FA}}}_{\mathrm{cont}}^{\mathrm{meas}}=0.26$ before correction (Fig. 6a) to ${\bar{\Delta \text{FA}}}_{\mathrm{cont}}^{\text{c}\text{o}\text{r}2}=0.29$, after correcting for the LPF matrix (Fig. 6b). This effect was mainly driven by the fact that the FA values in GM were smaller for the corrected FA maps than for the measured FA maps (*Δ*FA > 0.05, see Figs. 6e and 6g). The effect of the correction was particularly evident in small FA regions in the frontal GM (blue regions in Fig. 6c), corresponding to regions in which the FA of the LPF ellipsoid was largest (red regions in Fig. 6d).

## Discussion

We have introduced a linear model that estimates the local perturbation field (LPF) in DTI data from DW images of a water phantom. Simulations showed that the model is valid and robust. Moreover, we introduced the concept of an LPF ellipsoid which allows us to predict and better understand the effect of the LPFs on human DTI data. In particular, we used the LPF ellipsoid to reveal a characteristic artefact caused by LPFs in FA maps: the contrast between GM and WM is reduced, because small FA values are biased towards greater values. Finally, we estimated the LPFs for three different scanners and showed that they varied strongly in amplitude and spatial distribution. Our proposed model was capable of correcting phantom and human DTI data. After correction, the agreement between DTI data from different scanners and protocols was improved. The accuracy of the MD maps was increased. Furthermore, the contrast between GM and WM in FA maps was improved.

### The effect of LPFs on human brain DTI

The effect of LPFs on human DTI data is difficult to intuitively predict even if the LPF is known as pointed out by Bammer et al. (2003), since diffusion in the human brain is heterogeneous. We specifically investigated the effect of LPFs during diffusion weighting on MD and FA images. Our results showed that in perturbed FA maps small FA values within GM were biased towards higher values, whereas the bias of FA within WM did not show a consistent trend (Fig. 6g). This behaviour decreased the contrast between GM and WM and reduced the contrast-to-noise ratio in biased FA maps prior to correction. These observations are in accordance with previous studies reporting that small FA values are particularly susceptible to perturbations (e.g. Pierpaoli et al., 1996; Skare et al., 2000; Bammer et al., 2003). Furthermore, wide-spread bias in MD was identified and could be accurately corrected by the proposed model (Fig. 5). If the brain is scanned in different positions, e.g. by imperfect repositioning in longitudinal DTI studies, then the spatial variation in the LPFs will cause different bias and decrease intra-subject reproducibility (Fig. 5e). The correction improves the reproducibility of MD values (Fig. 5f). Accordingly, the proposed correction method might be particularly important in group and/or longitudinal studies, where variation in the subject positioning might occur.

### The LPF of different scanners

In multi-centre studies, the reproducibility and comparability of DTI data are critical issues. The LPF is a measure to assess the similarity of DTI data acquired on MRI systems with different technical specifications. An important advantage of our approach of measuring the LPFs is the fact that it enables researchers to apply the correction (as introduced by (Bammer et al., 2003) even if they do not have access to the manufacturer's information about the MRI scanner's gradient nonuniformities. Moreover, it does not only correct for the effects of gradient nonuniformities but also for LPFs due to EC fields, mis-calibration of the gradient amplitude, concomitant field terms, and cross-terms between diffusion gradients and imaging gradients.

The LPFs and their sources strongly vary between scanners. While we expected the gradient nonuniformities to be always one major source of LPFs (Bammer et al., 2003), the cross-terms depend on the amplitude and timing of the employed gradients (Neeman et al., 1995). Gradient mis-calibration can be a few percent in magnitude (Nagy et al., 2007). In human MRI scanners EC fields of 0.005% (Reese et al., 2003) up to 0.5% (Wilm et al., 2011) of the applied diffusion-weighting gradient have been measured during the readout and even higher EC fields might be possible for animal or non-clinical MRI scanners.

We measured the LPFs for three different scanners and showed that they vary by one order of magnitude (Fig. 2). All DTI data sets reflected the isotropic diffusion in a water phantom better if the measured diffusion tensor was corrected for LPFs (Fig. 3). We also observed that the correction was effective for in-vivo data (regions with *Δ*MD ≥ 0.1 × 10^− 3^ mm/*s*^2^ and *Δ*FA ≥ 0.05) for DTI2 and DTI3 (Figs. 5 and 6), whereas it did not significantly change the results for DTI1. This is probably due to the fact that the amplitude of the LPF of DTI1 is much smaller than those of DTI2 and DTI3, and thus the effect of correction might be masked by noise (Fig. 3a). In accordance with Bammer et al. (2003), we showed that the correction was better if the whole LPF matrix was used instead of only its diagonal elements (Figs. 3 and 4). Finally, we showed that the correction increased the consistency of data collected at different sites (Fig. 4). Thus, the proposed correction will facilitate multi-centre DTI studies relying on optimal comparability of datasets.

### LPF ellipsoid $L^{\Sigma }$

It is important to identify regions, which may be affected by LPFs. For this purpose, we introduced a simple linear approximation of the perturbed diffusion tensor in the isotropic-diffusion limit—the LPF ellipsoid (Eq. (7)). We showed that the trace (or FA map) can be calculated from the LPF ellipsoid to identify regions within the perturbed diffusion MD (or FA map) of the diffusion tensor, which are most affected (Figs. 5 and 6). For brevity, we reported on the effect of LPFs on MD and FA maps only. However, perturbed regions within other types of human DTI summary maps, e.g. the parallel and perpendicular diffusivity or the principal axis of diffusion (Bammer et al., 2003; Nagy et al., 2007), can also be delineated using a corresponding map calculated from the LPF ellipsoid.

### Measuring the LPFs: robustness and validity

Similar to the study of Nagy et al. (2007, 2009) we used DTI measurements of a water phantom to estimate LPFs. We used a tensor model to condense information about the LPF from individual DW images into a data perturbation LPF ellipsoid $L^{\Sigma }$ (which is linearly related to the LPF matrix *Σ*). The tensor formalism and the indices derived from it (e.g., trace) are known to be more robust than using single DW images (Basser et al., 1994). We tested the robustness of our model with respect to variation in LPFs and noise. In particular, we showed that our model was performing robustly for an SNR of 10 and a signal drop to 1/5 relative to the non-DW reference image—a typical minimal SNR on modern 3T MRI hardware. For SNR levels below 10, the robustness of the LPF estimation needs to be further assessed, and it may also be necessary to account for non-Gaussian noise distribution (Triantafyllou et al., 2011). Using Monte Carlo simulations, we showed that the proposed model was capable of estimating the LPF matrix robustly (Fig. 1).

### Methodological considerations

DTI indices are often regarded protocol independent, quantitative, and comparable between different scanners (Tofts, 2003). However, in general they are not strictly independent of the acquisition protocol and quantitative, because the simple DTI model is only valid for Gaussian diffusion (Tofts, 2003). The diffusion measured in the human brain can be non-Gaussian (especially at higher *b*-values (e.g. Tuch et al., 2002)) and thus depend on the protocol parameters. Moreover physiological and instrumental artefacts can bias DTI indices (e.g. Mohammadi et al., accepted for publication). Our results showed that the correction of DTI data from different scanners with different scanning protocols decreases differences and improves the agreement between them (Fig. 4). In general, various different scanning protocols can be used for the LPF correction method as long as the DTI water phantom calibration data are available. Residual FA differences, which were still present after correction (Fig. 3c), might well be explained by the different acquisition protocols and violation of the simple DTI model (Table 1).

A limited performance of the correction method for specific data sets might be due to the following reasons: (a) the effectiveness of the method will depend on the basis function used for spatial modelling (Eq. (B.3)). We used 3rd-order spherical harmonics to model the spatial dependence of the LPFs, which was successfully used in previous studies to describe eddy-current and gradient nonuniformity effects (Bammer et al., 2003; Wilm et al., 2011). However, the rather high residual anisotropy in the corrected DTI3 data set (Fig. 3 and Fig. 4) might indicate that the spatial distribution is not fully modelled for this particular scanner. (b) Non-linear relations between effective and expected diffusion-weighting gradients (e.g. miscalibration between ± *x* gradients as reported by Nagy et al. (2007)) cannot be fully corrected by linearised models like ours or Bammer et al.'s (2003). (c) Artefacts in the water phantom measurements that are not described by LPFs, e.g., vibration or ghosting artefacts, might be another potential confound. However, we believe those artefacts are unlikely to limit the performance of the estimation, since a strength of the LPF matrix approach is that it is model driven and artefacts that are not proportional to the diffusion-weighting gradient direction (e.g. vibration artefacts (Gallichan et al., 2010; Mohammadi et al., accepted for publication) or time-dependent flow effects (Callaghan, 1998) will not bias the LPF matrix estimation but only increase the noise on the estimates (i.e. the residual error of the model fit). We used a weighted-least square approach to down-weight those artefacts when estimating the LPF matrix (Eq. (B.6)).

In this study, we used the ST sequence to acquire DTI data. However, the correction can in principle also be applied to DTI data acquired with the twice-refocusing spin-echo sequence (Reese et al., 2003). Although the model is versatile, the correct estimated LPF values are not universally applicable and should always be based on the specific acquisition used for acquiring human data. For example, the angulation of the scan planes might affect the LPF matrix, e.g., due to interaction between imaging and diffusion-weighting gradients (Neeman et al., 1995). Therefore, the same slice prescription was used for the phantom calibration scan and human DTI data acquisition.

It is known that LPFs can perturb the orientation of the diffusion tensor (Bammer et al., 2003) and affect tractography results (Nagy et al., 2007). Future studies may look specifically at tractography results, before and after correction of LPFs.

Our proposed model uses a linear approximation, for which the estimated LPF matrix is symmetric (see Eq. (7)). Our model does not estimate the non-symmetric part of the LPF matrix, since we showed analytically that the perturbations of the diffusion tensor are sufficiently corrected by the symmetric part of the LPF matrix (Eq. (8)).

## Conclusion

We have presented a simple model to assess and correct the effect of LPFs during diffusion weighting. The proposed model can be used to improve the sensitivity and reduce artefacts in DTI data. This is of particular importance in studies which search for FA or MD differences of few percent between controls and patients (Deppe et al., 2008), or where the diffusion in grey matter is measured (Heidemann et al., 2010; Nagy et al., 2011). The correction improves the reproducibility and comparability of DTI data sets, in particular in multi-centre or longitudinal studies. The proposed approximation of the perturbed diffusion tensor in the isotropic-diffusion limit provides clear and straightforward insights into how diffusion studies are affected by LPFs. The movement toward higher gradient amplitudes in DTI at higher fields (Lohmann et al., 2010; Lutzkendorf et al., 2009) and for in-vivo quantification of axonal diameters (Alexander, 2008; Assaf et al., 2008) will make appropriate corrections of LPFs even more important.

## Figures and Tables

**Table 1:** Summary of MRI protocol details.

Acquisition hardware	DW image direction / b-value [s/mm^2^]	Non-DW images (b = 0)	TE [ms]	TR [s]	Max diffusion-weighting gradient [mT/m]	Partial Fourier (PF)/asymmetric echo (AE)	Duration of DW gradients (Δ [ms] /δ [ms])	Matrix (in-plane res. [mm^2^])	Slices (thickness [mm])	Field of view [mm]	Parallel acq. acc.	Cardiac gating
DTI1	60 / 1000	6	81	9	36	1/4 (AE)	31 / 21	96^2^ (2.3^2^)	60 (2.3)	220	No	No
DTI2	61 / 1000	6	98	14	55	7/8 (PF)	50 / 10	96^2^ (2.3^2^)	72 (2.3)	220	No	No
DTI3	52 / 1200	6	73	Variable^a^	40	5/6 (PF)	29 / 21	96^2^^b^ (1.9^2^)	60 (2.4)	243	Yes / factor 2	Yes

a Depending on heartrate.

b Reconstructed to matrix of 128^2^.

## Appendix A First**-**order approximation in the LPF

It follows from Eqs. (2) and (5) that the elements of the perturbed B matrix are given by (written with the Einstein summation convention):

(A.1) B∗kl=gk∗gk∗=gk+ϵkmgm2fork=l≈gk2+2gkϵkmgm,

(A.2) B∗kl=gk∗gl∗=gk+εkmgmgl+ϵlngnfork≠l≈gkgl+gkϵlngn+glϵkmgm

where we assumed that the LPF matrix elements *ε*_*kl*_ are small (*ε*_*kl*_ ≪ 1 for *k*, *l* = 1, …, 3). Using this approximation and reordering the sum in Eq. (3) ( ${\text{ADC}}_{i}^{*}=\sum_{kl=1}^{3}\;B_{\mathrm{ij}}^{*}D_{\mathrm{ij}})$ allows us to express the perturbed **B** matrix as:

(A.3) $$B^{*}\approx B_{i}+2\Sigma^{+}B_{i}\text{,}$$

where *Σ*^+^ is the symmetrical part of the LPF matrix *Σ*^+^ = (*Σ*^T^ + *Σ*)/2.

## Appendix B Estimating the LPF matrix elements

Step 1 For a given voxel, the elements of the $L^{\Sigma }$ matrix can be estimated from water phantom DTI data using a least square estimation (Basser et al., 1994):
  (B.1) a^=XTX−1XTy,
  where the data vector, *y*, the model matrix, X, and the model vector, $\hat{a}$, are given by:
  (B.2) y=1DwADC1∗,…,ADCNDG∗T,X=x1∗,…,xNDG∗,withxi∗=g1,i2,g2,i2,g3,i2,2g1,ig2,i,2g1,ig3,i,2g2,ig3,i,a^=L^(11)Σ,L^(22)Σ,L^(33)Σ,L^(12)Σ,L^(13)Σ,L^(23)ΣT,
  and *g*_*i*_ = (*g*_1, *i*_, *g*_2, *i*_, *g*_3, *i*_) is the *i*-th diffusion gradient vector, ${\hat{L}}_{\left(ij\right)}^{\Sigma }$ are the elements of the estimated LPF ellipsoid $L^{\Sigma }$ (Eq. (10)). Finally, the residual error is given by: $\sigma =y-X\hat{a}$. For simplicity we will omit the hat-symbol in the estimated $L^{\Sigma }$ matrix elements ${\hat{L}}_{\left(ij\right)}^{\Sigma }\to L_{\left(ij\right)}^{\Sigma }$.

Step 2 The LPFs might reasonably be expected to be a smooth function of position *r*. We make use of the smooth nature of the LPFs by using a series of spherical harmonics up to the order *M*_*l*_ (Bammer et al., 2003; Wilm et al., 2011) to estimate the spatial dependency of the elements of the LPF matrix *ϵ*_(*ij*)_(*r*):
  (B.3) ε(ij)(r)≈∑l=0Mlαl(ij)Pl(r)
  with *P*_*l*_(*r*) being the spherical harmonics of the order *l* and *α*_*l*_^(*ij*)^ the corresponding coefficients (Wilm et al., 2011).
  To estimate the coefficients of the spherical harmonics *α*_*l*_^(*ij*)^, we used as the data vector the LPF ellipsoid elements *L*_(*ij*)_^*Σ*^ and their linear relation to the LPF matrix elements (Eq. (7)). Then, for the *ij*-th element of the $L^{\Sigma }$ matrix, *L*_(*ij*)_^*Σ*^(*n*), Eq. (B.3) can be rewritten as a matrix equation of discretised space *n* = 1, …, *N*_*r*_, reordered as an array with length *N*_*r*_ = *N*_*x*_ × *N*_*y*_ × *N*_*z*_:
  (B.4) $$\varepsilon_{\left(ij\right)}=P\alpha^{\left(ij\right)}\text{,}$$
  where the model vector elements being ***α***^(*ij*)^ = [*α*_1_^(*ij*)^, …, *α*_*M*_*l*__^(*ij*)^]^T^, the elements of P being *P*_*i*_(*j*) (with *i* = 1, …, *M*_*l*_ and *j* = 1, …, *N*_*r*_), and the data vector elements being:
  (B.5) εii(n)=1−L(ii)Σ(n)2fori=1,…,3εij(n)+=L(ij)Σ(n)2fori≠jandi=1,…,3andj=1,…,3

We used a weighted least-square solution of the matrix Eq. (B.4):

(B.6) $$\alpha^{\left(ij\right)}={\left(P^{\text{T}}WP\right)}^{-1}P^{\text{T}}W\epsilon^{\left(ij\right)}$$

The elements of the diagonal weighting matrix *w*_*nn*_ are functions of the root-mean-square of the residual error *σ*(*n*) of the fit of the $L^{\Sigma }$ matrix (Eq. (B.1)) at voxel position *n*:

(B.7) $$w_{\mathrm{nn}}=\frac{1}{1+{\hat{x}}^{2}}\;\text{with}\;\hat{x}=\frac{\sqrt{\langle \sigma {\left(n\right)}^{2}\rangle }}{\frac{1}{N_{r}}\sum_{n=1}^{N_{r}}\sqrt{\langle \sigma {\left(n\right)}^{2}\rangle }}\text{.}$$
